# Recent advances in the biomolecules mediated synthesis of nanoclusters for food safety analysis

**DOI:** 10.1016/j.heliyon.2023.e15655

**Published:** 2023-04-20

**Authors:** Devaraj Sabarinathan, Arumugam Selva Sharma, Marimuthu Murugavelu, Balakrishnan Kirubasankar, Indhiradevi Balusamy, Zhang Han, Huanhuan Li, Quansheng Chen

**Affiliations:** a School of Food and Biological Engineering, Jiangsu University, Zhenjiang, PR China; b Department of Energy Science, Sungkyunkwan University, Suwon, South Korea; c Department of Pediatrics, Kanchi Kamakoti Childs Trust Hospital, Chennai, India; d Athenese Dx Pvt Ltd, Chennai, Tamilnadu, India

**Keywords:** Metal nanocluster, Synthesis, Microbes, Pesticides, Metal ions, Drugs

## Abstract

The development of nanoclusters based on incorporating biomolecules like proteins, lipids, enzymes, DNA, surfactants, and chemical stabilizers creates a stable and high fluorescence bio-sensors promising future due to their high sensitivity, high level of detection and better selectivity. This review addresses a comprehensive and systematic overview of the recent development in synthesizing metal nanocluster by various strategized synthesis techniques. Significantly, the application of nanometal clusters for the detection of various food contaminants such as microorganisms, antibodies, drugs, pesticides, metal contaminants, amino acids, and other food flavors have been discussed briefly concerning the detection techniques, sensitivity, selectivity, and lower limit of detection. The review further gives a brief account on the future prospects in the synthesis of novel metal nanocluster-based biosensors, and their advantages, shortcomings, and potential perspectives toward their application in the field of food safety analysis.

## 1 Introduction

In recent years, concerns related to food safety issues are on the raise due to the contamination of food stuffs with microbes, metal ions, pesticides and organic chemicals. The consumption of unsafe food causes 200 different types of diseases that affect infants to elders, from diarrhea to cancers. Almost 1 in 10 people are falling ill after eating the contaminated food each year, resulting in 420 000 deaths with the loss of 33 million people healthy life years in an estimate of at least 600 million population. According to a report, 40% of children under five years of age are suffering from food-borne related disease out of these an estimated 125 000 deaths were reported every year by World Health Organization, 2020. Most of these infections are caused by microbes such as *Salmonella*, *E. coli*, Listeria, Vibrio, Viruses, Parasites and chemicals like naturally occurring toxins, persistent organic pollutants and heavy metals [1]. Therefore, it is necessary to find a cost effective and real time sensing strategy to accurately detect trace levels of various toxic chemicals, metal cations, anions, drugs, amino acids and pesticides in various environmental, biological and food samples. These contaminants are easily identified by a variety of detection strategies including GC-MS, HPLC and other biological assays.

**Fig. 1:**
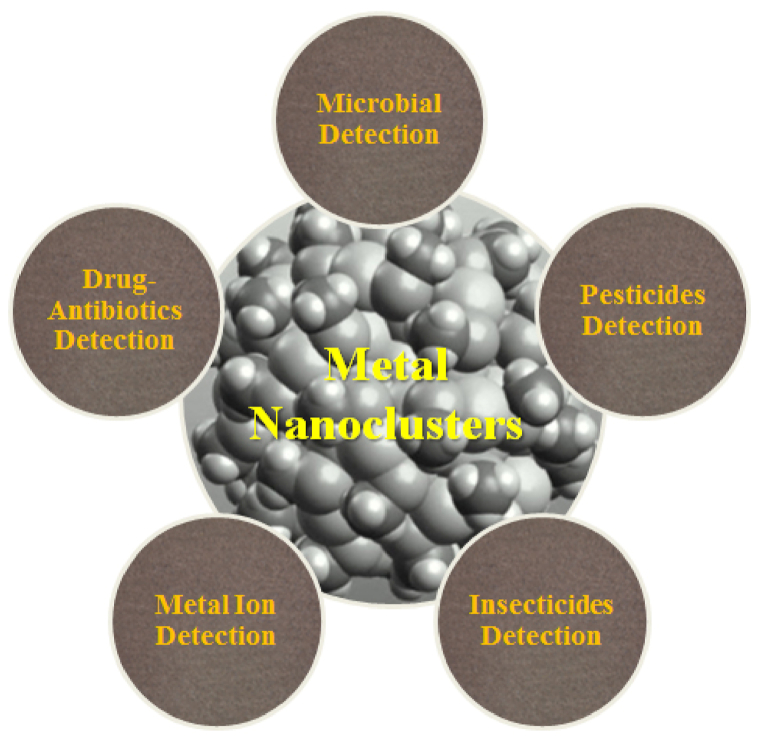
Representation of MNCs application.

**Fig. 2:**
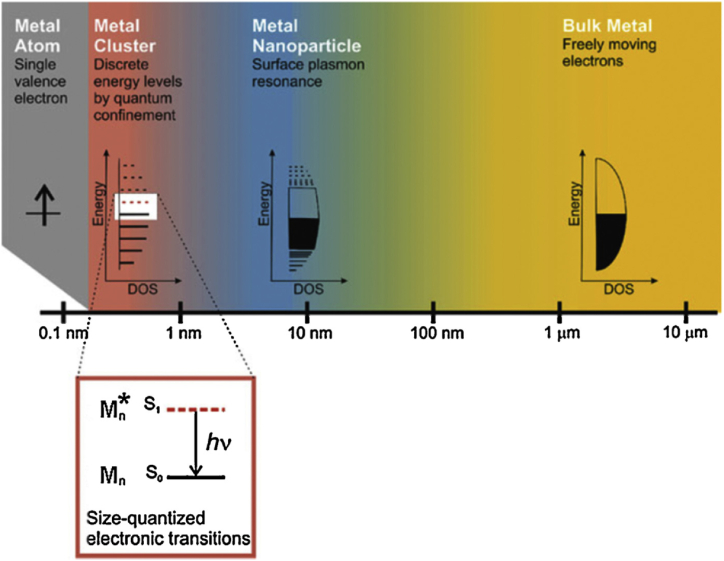
MNCs bridge and detections. Reprinted from Ref. [4] with permission by Springer-Verlag.

Fluorescent metal nanoclusters (MNCs) are emerging fluorophores have attracted immense interest from researchers because of their excellent features such as biocompatibility, photostability, sub-nanometer size, distinctive luminous capabilities and ease of synthesis (Fig. 1). MNCs are the missing link between metal atoms (which have different optical properties) and nanoparticles (NPs) (which have plasmons) and exhibit molecule-like behavior. Metals' electrical and optical characteristics are strongly influenced by their size, especially in the nanoscale range. The conduction band in bulk metal has no energy gap separating it from the valence band. Therefore, electrons do not encounter a barrier and can travel freely. The mean free path of electron determines the scattering of electrons. When the size of the metal NPs is comparable to or smaller than the electron mean free path, the electron's motion is constrained by the size of the NPs, and interaction is expected to occur mainly with the surface, resulting in surface plasmon resonance (SPR) band [2]. In metal NCs, the size of metals is further reduced around 1 nm or less, down to a few atoms, and the continuous band structure is broken into discrete energy levels. Nanoclusters (NCs) are non conductive and plasmonic. Interaction with light still exists, but it occurs through electronic transitions between energy levels, similar to organic dye molecules, resulting in light absorption and emission. As fluorescent probes in fluorescent biosensing and bio-imaging, they have the same optical property as quantum dots and fluorophores. Fluorescent MNCs are crucial for recognition of metal atoms from NPs via optical properties and Plasmon effects [2] (Fig. 2). These optical properties lead to a variety of fluorescent probes that have been used in biological imaging, bio-sensing of metal ions, insecticides, and other applications [3].

Researchers have paid close attention to the unique physical, chemical, electrical, and optical properties of NCs. Nowadays, NCs are synthesized by various methods in different sizes with fluorescence properties that can be tuned from the UV to the near range – IR region. NCs are capped with biomolecules such as proteins, oligonucleotides, enzymes, and peptides because they have high luminescence quantum yields, nontoxic, tunable luminescence features and biocompatible nature. The advantages and the development of biosensing and imaging applications are based on the unique features of optical probes [5,6]. The synthesis of fluorescent NCs and their application for the development of sensor platforms are the subject of extensive research activities.

Food, biomedical, forensic, and environmental materials are contaminated by several chemical and biological agents. Fundamental biology, chemistry, and materials science are required to develop advanced technologies with sensitive, low-cost sensors [ [7,8]]. However, the technology possesses several challenges requiring sophisticated instrumentation, modification of existing techniques, and complex sample handling. Therefore, the detection of toxic chemicals and biomolecules in a simple, label-free and cost-effective manner using quantitative and qualitative methods has become more important. Fluorescent MNCs have gained attention for rapid, selective and sensitive detection of various toxic chemicals, biomolecules and the environmental samples. Many existing fluorescent probes are mainly based on organic fluorophores, quantum dots and fluorescent proteins [8,9]. The various organic fluorophores differ in their chemical structure spectral properties, and susceptibility to photobleaching, which limits their potential applications in the respective field. Quantum dots appear larger in physical, photostable, which may liberate hindrance in time of binding, which may compromise their use for *in vivo* applications and toxic nature. MNCs appear in ultra-small size, low toxicity, possess good biocompatibility, low cost with excellent photostability properties compared to over other organic dye molecules and semiconductor quantum dots [8,10]. The origin of the fluorescence, stability, binding capacity of the various functional groups, and biocompatibility properties in NCs are related to the ligand or templates. Biomolecules like proteins, thiols, peptides, enzymes, lipids, DNA, Polymers and dendrimers are used as a stabilizer to prepare noble MNCs with several advantages. For example, the emergence of fluorescent nanomaterials, especially fluorescent MNCs, has had a profound influence on optoelectronic devices, bio-marker, and biosensors. In fact, MNCs showed great promise for a number of applications, including electronics, chemistry, biology, pharmaceuticals, and biosensing. MNCs-based biosensors found applications in a variety of domains, including healthcare and food safety, offering quick and low-cost analysis of a variety of target molecules. There are number of review articles on the synthesis, properties and versatile applications of MNCs [11,12].

Various strategies have been used to detect cations and anions at trace levels, including atomic absorption spectrometry (AAS), inductively coupled plasma mass spectrometry (ICP-MS), electron paramagnetic resonance (EPR), and synchrotron radiation X-ray spectrometry (SRXRS) [13]. However, these detection methods are high cost, time consuming, need sophisticated instruments, and sample pretreatment of samples. In recent years, in order to overcome this issue, fluorometric detection techniques have been touted as a potential alternative for the detection of various analytes with high specificity, high sensitivity, and reproducibility. Moreover, in fluorometric detection, the monitoring of biological samples in real-time reduces the risk of contamination and time consumption. Further, semiconductor quantum dots and organic dyes are used for sensing of toxic chemicals and bioimaging applications. However, they possess many limitations, such as less photostability and high toxic nature. Compared to quantum dots, MNCs have distinct advantages and are widely used for sensing and bioimaging applications. For instance, luminescence sensors, electrochemical sensors, optical biosensors, electrochemical biosensors, and photoelectrochemical biosensors are examples of MNCs-based sensors. To the best of our knowledge, no systematic study of MNCs-based fluorescence sensing applications for food quality analysis has been published yet. Although several sensing methodologies utilizing MNCs have been developed, only a few studies have focused on the detection of hazardous compounds and physiologically essential molecules for food safety applications. This review article provides an overview of recent advances in the synthesis and photophysical properties of MNCs and their application in food safety analysis.

## 2 Synthesis of metal nanoclusters

**Fig. 3:**
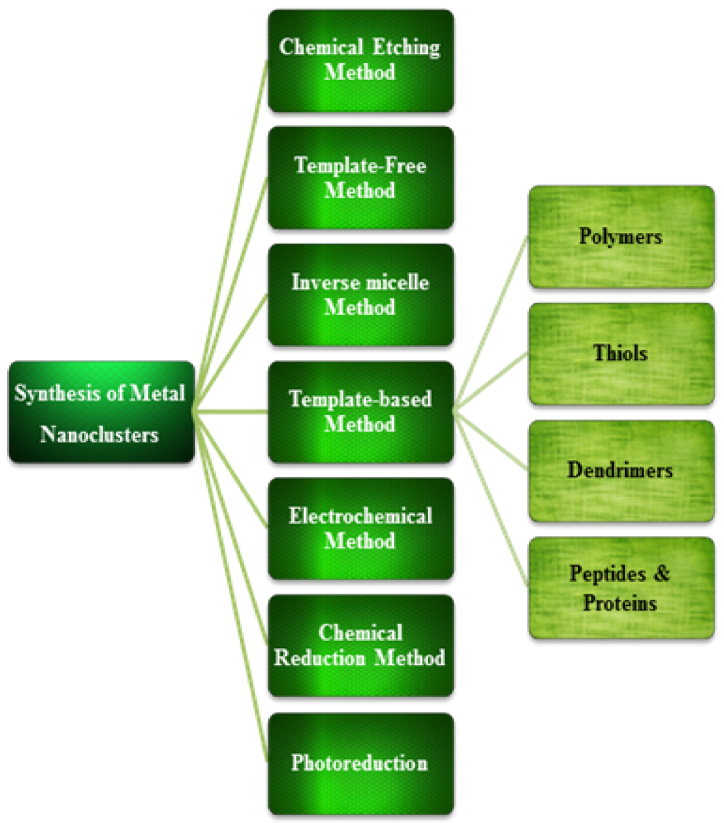
Schematic representation for synthesis of MNCs.

**Fig. 4:**
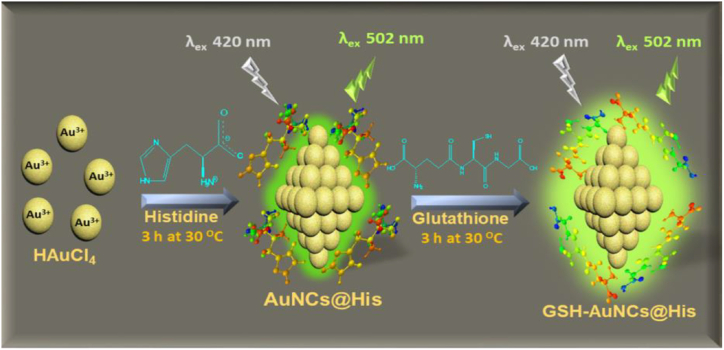
Illustration of GSH binding with AuNCs modified with Histidine. Copyright from Ref. [16].

The structure, size, and surface properties of NCs depend upon the concentration of templates or ligands, reducing agents, metal ions and temperature. Proteins, peptides, polymers, and chemicals act as capping agents to produce highly stable fluorescent nanocluster. The origin of the fluorescence, the sensitivity of NCs with different spectral regions depends on the stabilizer. NCs are mainly synthesized based upon photoreduction, chemical reduction and templates (Fig. 3). The Ag^+^, Au^3+^ and Cu^2+^metal ions are reduced in the presence of the stabilizers there by resulting in the formation of fluorescent NCs. These processes proved that proteins itself acted as effective reducing and capping agents and did not require any additional reducing agents. Another method for creating NCs is to employ small molecules such as thiol compounds in an alkaline solution, as well as template-free techniques and the electrochemical methods for etching bigger NPS (2–4 nm). The chemical reduction method was the most commonly used technique to obtain MNCs. For instance, AuNCs are generally synthesized by reducing Au^3+^ with capping and reducing agents [12]. Thiol compounds are used as a capping agent to minimize the complicated bonding character of Au–S through Au atoms/ions. Reducing agents are typically used to synthesize NCs in the presence of thiol compounds such as tetrakis-(hydroxymethyl), phosphonium chloride (THPC), and sodium borohydride, (NaBH_4_). It was previously reported that when NaBH_4_ is utilized as a reducing agent in the presence of glutathione (GSH), glutathione-stabilized AuNCs (GSH-AuNCs) are formed from Au^3+^ [14]. Different sizes of GSH−AuNCs emit at the emission wavelengths after separation and purification with 0.1 percentages of quantum yields (QYs) (Fig. 4). Stabilized AuNCs are made by utilizing different thiols such as polyethylene glycol attached lipoic acid, phenylethyl thiolate, tiopronin, and thiolate cyclodextrin by altering an identical technique. When the molar ratio of thiol to Au ions is increased, the size and QY of thiol-stabilized AuNCs usually decrease. However, the majority of the thiol-stabilized AuNCs produced had low QYs. On the other hand, GSH is used as a reducing agent in the production of NCs. GSH acted as a potent capping agent and a weak reducing agent which leads to inadequate reduction of Au^3+^ to Au^2+^ to produce the Au NC core, which was stabilized with thiolate-Au^+^ complexes. Fluorescent Au @ Au^+^- thiolate core-shell NCs were synthesized with low thiol Au (1.5:1) by the thiolate-based complex's generated Au cores at controlled aggregation with the QY of 15% [15]. In other hand, synthesized CuNCs from CuCl_2_ by a simple chemical reduction method using N_2_H_4_ H_2_O as a reducing agent and BSA as a stabilizer. The light-yellow color indicated the formation of CuNCs.

### 2.1 Template-based synthesis

Template-based synthesis is an effective way to synthesis MNCs. Many templates are used to synthesize MNCs such as polymer, thiols, molecular sieves, peptides and proteins, DNA oligonucleotides, dendrimers, polyelectrolytes etc. The core size and size distribution of the MNCs can be controlled by the template’s method [17].

#### 2.1.1 Polymers

Many MNCs are synthesized by the polymer-based template method. For instances, polymer with carboxylic acid and alcoholic groups is used as a stabilizing agent. By varying the polymer to metal ratio, MNCs shape, size and fluorescent properties are tuned easily. Researchers reported the preparation of Ag NCs by utilizing Poly methacrylic acid (PMAA) as a template by reducing the AgNO_3_ precursors under ultraviolet irradiation. It has been reported that when 60 mg/mL AgNO_3_ and 10 mg/mL PMAA solution were exposed to UVA for 60 min, the solution turned pink color which indicates the formation of AgNCs [18].

Photoreduction methods are used in the preparation of MNCs by preventing hazardous inorganic reducing agents [17]. The MNCs size and QYs are mainly dependent on the nature of polymers used in the synthesis and can be varied using the molar ratio of polymer and metal ions. PVP was used as a template for the preparation of CuNCs by a facile chemical method 0.2 g PVP, 300 μL L-ascorbic acid, 100 μL of CuCl_2,_ and 10 mL of double distilled water added into a 100 mL of Carousel 6 Plus solution which was then kept under 90 °C for 21 h to the yield PVP capped CuNCs [19]. [20] Aparna et al., synthesized CuNCs using polyethyleneimine (PEI) as a capping agent or stabilizing agent. CuSO_4_ and ascorbic acid were mixed with 0.094 g mL^−1^ PEI solution to form a colloidal suspension. This reaction mixture was exposed to microwave irradiation for 20s to obtain CuNCs-PEI [21]. Hu et al., used poly (sodium-p-styrene sulfonate) (PSS) as a template for silver nanocluster synthesis. In a typical synthesis they used 17 mg of AgNO_3_, 14.9 mg of D-penicillamine and 50 mL of double distilled water mixed with 150 mg of PSS, and the complexes were exposed to UV-lamp of 300W for 25 min to obtain the PSS-DPA-AgNCs (D-Penicillamine stabilized argentum nanoclusters) (Table 3) [22]. Lewis et al., used 5 mL of polyethylene glycol (PEG) with 50 mL of 1 mM HAuCl_3_ and 4 mL of trisodium citrate into the solution. A color change from black to red indicated the formation of AuNCs-PEG.

#### 2.1.2 Thiols

The thiols-based template contains a small stabilizer molecule which provides a strong interaction for synthesizing MNCs. Various thiols have been used to synthesize MNCs like glutathione, 3-mercaptopropionic acid, thiolate-cyclodextrin and tiopronin [35]. X. Liu and coworkers prepared CuNCs by using 2-mercapto-5-n-polypyrimidine (MPP) as a protecting thiol group and using NaBH_4_ as a reducing agent. The chemical reduction method is the most commonly used technique to obtain MNCs. For example, the most typical process for the production of AuNCs is the reduction of Au^+^ from Au^3+^ to obtain Au^0^. Thiol groups, as capping agents, are known to bind strongly to gold surfaces due to sulphur bonding. To prepare NCs, thiol reducing agents such sodium borohydride (NaBH_4_) and tetrakis-(hydroxymethyl) phosphonium chloride (THPC) are commonly utilized. It has been reported in the presence of reducing agent NaBH_4;_ glutathione (GSH) stabilized AuNCs (GSH−AuNCs) have been prepared from Au^3+^ions. GSH−AuNCs with <0.1% of quantum yields (QYs) emit at emission wavelength obtained in various sizes after the purification and separation process. AuNCs are stabilized using many other thiols such as phenylethyl thiolate; polyethylene glycol appended lipoic acid, thiolate cyclodextrin, and tiopronin following the same methods of preparation. The concentration of molar ratio of thiol increases with Au ions, resulting in smaller size of Au ions with lower QY. The prepared thiol stabilized AuNCs have consistently low QYs. GSH, which works as a capping and reducing agent, is commonly used for fluorescent NCs. AuNC core was produced under neutral conditions by the semi-reduction of Au^3+^, which was stabilized by monolayer thiolate-Au^+^ complexes. GSH works as a weak reducing agent with a QY of 15% at fluorescent Au@Au^+^- thiolate core-shell NCs, which are synthesized by controlled aggregation of thiolate and Au^+^ complex, at lower molar concentrations of thiol-Au (1.5:1). Glutathione was used to prepare CuNCs by the following method: 50 mM of CuCl_2_ was mixed with 0.21 mM of glutathione under the stirring condition to form copper thiolate complexes. Then the colloidal suspension was centrifuged and dialyzed to obtain CuNCs with a quantum yield of 8.6% [36]. [37] Gayen et al., used 10 mM of HAuCl_4_ and 0.11 M of MPA (Mercaptopropionic acid) and heated at 50 °C for 2 min. Then the solution is illuminated under a UV trans illuminator at 305 nm; on excitation, a bright luminescence was observed. MPA-AuNCs were synthesized using MPA and NaOH [38]. [39] Nath et al., synthesized AuNCs using 2-mercapto-4-methyl-5-thiazoleacetic acid (MMT). In a typical synthesis, 2.5 mg of MMT, 0.1 M HAuCl_4_, 10 mL of citrate and 1.0 mL of NaOH were mixed at room temperature and stirred for 8 h to obtain MMT-AuNCs.

#### 2.1.3 Dendrimers

Dendrimers are used to prepare small MNCs. A low yield of MNCs was obtained from this method [40]. Zheng et al. prepared stabilized AuNCs by dendrimers with a high yield of 42%. By varying the concentration of (poly (amidoamine) dendrimers) PAMAM/Au, produced AuNCs with ranging emission colors from UV to near-infrared region.

#### 2.1.4 Peptides and proteins

Biological macromolecules like peptides and proteins were also used as a template for the synthesis of MNCs. The macromolecules provided high binding sites to reduce metal precursors, which offered small MNCs. Several proteins such as human serum protein, insulin, lysozymes proteins, Ovalbumin etc., were used as templates. Some enzymes were capable of catalytic activity towards the production of MNCs [41]. Bhamore et al., synthesized AuNCs using amylase solution. They used 17 mg of HAuCl_4_, 0.25 g of amylase and 2 mL of NaOH to obtain amylase capped-AuNCs. CuNCs are prepared using bovine serum albumin (BSA) through a simple one-pot method [42]. CuSO_4_ (20 mM) was added to BSA solution (5 mL @ 15 mg/mL) with the addition of NaOH, the pH was maintained at 12 and the reaction mixture was incubated at 55 °C for 8 h to obtain fluorescent CuNCs. CuNCs from CuCl_2_ were synthesized by a simple chemical reduction method using hydrazine as a reducing agent and BSA as a stabilizer agent. The light-yellow color indicated the formation of CuNCs -carbon dots/CuNCs nanohybrid (CDs/CuNCs). CuNCs were made using a simple chemical reduction process with hydrazine as a reducing agent and BSA as a stabilizer agent, whereas carbon dots were made using a hydrothermal method [15]. Kalaiyarasan et al., first used chicken egg white (CEW) as a template for the preparation of AuNCs with the size of 2 nm and also generated a red fluorescence at 720 nm with excitation at 535 nm [43]. In a similar way Guo et al. [44], used CEW for the synthesis of AuNCs. Similarly [45], Yan et al., also synthesized AuNCs by microwave-assisted method using CEW. The CEW-AuNCs had a size of 3.56 nm, emitted red light at 667 nm, and had a quantum yield of 7.23%. Akyüz et al., synthesized CEW-AuNCs by using 2.5 mM HAuCl_4_ solution added with 25 mL of protein solution, to which 3.5 mL of NaOH solution is added and incubated at 37 °C for 20 h to obtain CEW-AuNCs [46]. Bhamore et al., produced AuNCs using Histidine (100 mM) and GSH (30 mM) from HAuCl_4_ precursor (10 mM) [16]. Chen et al., used ovalbumin for the synthesis of AuNCs: 10 mM HAuCl_4_, 50 mg/ml Oval albumin (OVA) and 0.35 mg/mL of ascorbic acid (AA) were mixed vigorously under stirring and pH of this solution is adjusted by adding 0.5 mL of NaOH solution. The resulting solution was incubated at 95 °C for 20 min to get AuNCs-OVA [47]. Bhamore et al., developed BSA-bromelain-AuNCs by adding an equal amount of 2.5 mL BSA and bromelain with 5.0 mL of HAuCl_4_ under constant stirring [48]. To this, 0.5 mL of NaOH was added and incubated at 37 °C for 10 h to obtain AuNCs-BSA. Serum protein BSA was used to prepare AuNCs by a facile chemical reduction method, 12.5 mL of HAuCl_4_, 1.25 mL of NaOH, 12.5 BSA, and 1.25 mL of MPA were mixed and incubated at 4 °C for 1hr to get BSA/MPA-AuNCs [49]. To make AuNCs, Cheng and his colleagues used soybean protein: 5 mL HAuCl4 and 5 mL soybean protein were combined with 1 mL NaOH which resulted in light yellow to pale brown colour indicated the creation of SP-AuNCs [50]. [51] Lu et al., prepared Lys-AuNCs by following method: 4 mM HAuCl_4_, 16 mg/mL of lysozyme solution and 1 M NaOH were added at 37 °C under continuous stirring for 8 h, where the light-yellow colored liquid changed to deep brown indicating the formation of Lys-AuNCs.

### 2.2 Chemical etching

Chemical etching offers a straight forward approach to prepare NCs from metallic NPs. For example, the ligand-induced etching method produces excess ligands used to prepare AuNCs from the Au NPs with 2–4 nm core size. THPC acts as a capping and reducing agent in preparing AuNCs using thiol ligands such as 11-mercaptoundecanoic acid (11-MUA) as an etching agent under alkaline conditions. 11-MUA causes a significant etching ability on the surface of Au atoms. At a high pH (>12.0), it creates strong coordination to form stable 11-MUA-Au complexes on the Au surface in each core of the shell, resulting in fluorescent capped AuNCs with a QY of 3.1% [52]. Different thiol compounds can be used to alter the size and optical characteristics of AuNCs. Alkane thiols act as ligands in the production of alkane thiol-bound AuNCs with varied chain lengths and emission wavelengths ranging from 501 to 613 nm QYs ranging from 0.0062 to 3.1%. The emission properties of 11-MUAAuNCs are tuned from 524 to 456 nm by changing the molar ratio of Ag^+^/Au3^+^ from 0 to 1.6. The variable fluorescent wavelength, long lifespan (>200 ns), and considerable Stokes shift are all features of these fluorescent AuNCs. (>100 nm) [52]. Sun et al., used 3:1 HNO_3_: HCl, 1% trisodium citrate and 0.075% NaBH_4_ for the chemical etching of HAuCl_4_ solution to obtain AuNCs of size 13 nm [53]. Mecker et al., developed AuNCs with sodium citrate and HAuCl_4_ under reflux for 15 min, the golden color solution turns into red wine color indicating the formation of AuNCs. Blue emission is seen in the prepared AuNCs, with QYs of 3.8%, 14.3%, and 20.1%, respectively [54].

### 2.3 Template-free method

The template-free methods were a simple, facile and effortless method for the production of MNCs. There is no extra substance, minimal post processing using the strong ionic or acid-alkali condition to obtained pure MNCs in this method [[55], [56], [57]].

### 2.4 Inverse micelle synthesis method

The inverse micelle synthesis technique was a liquid phase synthesis method. A droplet-like inverse micelle was used for this synthesis. It is an inexpensive, facile and simple technique. In this process, the cluster size was controlled by regulating the concentration of metal salt precursors and surfactant concentration [[58], [59], [60]].

### 2.5 Electrochemical synthesis method

The electrochemical synthesis method was a promising route for the preparation of MNCs. Here, the shape and size can be altered using the electrochemical parameters. In electrochemical synthesis anode acts as the metal source. By anodic dissolution or reduction at the cathodic surface, the MNCs were formed and balanced using surfactants in the electrolyte solution [35]. under electrochemical synthesis using Cu anode, CuNCs are made using Cu as an anode, Pt as a cathode and 0.1 M of tetrabutyl ammonium nitrate as an electrolyte solution and Ag/AgCl as a reference electrode.

### 2.6 Photo reduction

To eradicate the hazardous inorganic reducing materials like NaBH_4_, trisodium citrate etc. photoreduction methods have been utilized for the formation of MNCs. For example, MNCs with fluorescent characteristics, are produced by photoreduction of tridentate thioether terminated polymers such as poly (n-butyl methacrylate), poly (tetra-butyl methacrylate), and poly (methyl methacrylate). The size and yield of MNCs are determined by the nature of polymers, which can be changed by adjusting the molar concentration of polymer to metal ions [61,62].

## 3 Applications

### 3.1 Microbial detection

**Fig. 5:**
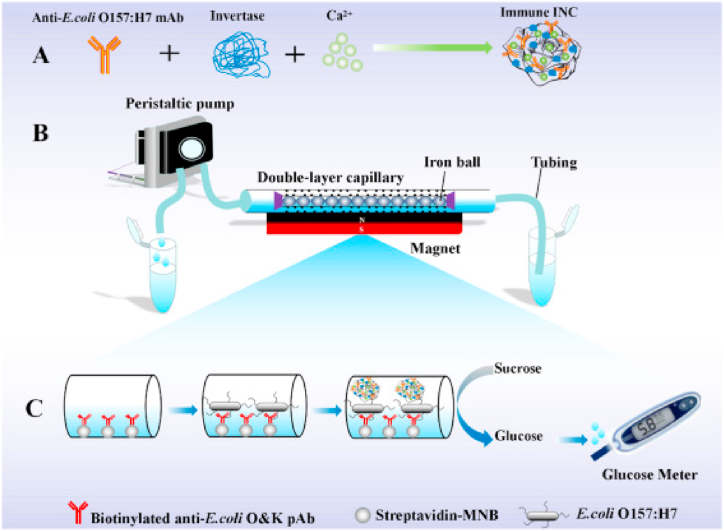
Schematic representation of biosensor based signal amplification for sensitive detection of *E.coli* 0157:H7Reprinted from Ref. [63].

Food and drinking water were contaminated by microorganisms like viruses, bacteria and protozoa. The microbes causing food spoilage were initially screened by biosensing with good selectivity and high sensitivity. Several MNCs -based sensors have been developed for the detection of pathogens [24]. Liu et al., detected *Listeria monocytogenes* by monitoring the visible color change of Ag NCs from blue to red in the concentration range of 10–10^6^ CFU. mL^−1^. The sensing assay exhibited a detection limit as low as 10 CFU mL^−1^. In other hand immune invertase- NCs (INCs) detected E.coli O157:H7 at concentrations ranging from 10^2^ to 10^7^ CFU mL^−1^, with the lowest detection at 79 CFU/mL, according to Huang et al. [63] (Fig. 5) (Table 3) [28]. Zhang et al., used DNA/Ag NCs to detect Ochratoxin's (Ap1) and Aflatoxin (Ap2) binding with mycotoxins. Since Ochratoxin (OTA) and Aflatoxin B_1_ (AFB_1_), the DNA/Ag NCs have set detection limits of 0.2 pg mL^−1^ and 0.3 pg mL^−1^, respectively [33]. Zhang et al., made a biosensor to detect *Salmonella* species using Fe–NCs with a lower detection limit of 14 CFU/mL and recovery as ∼105.0% in spiked chicken samples [32]. Subramaniyan et al., used phytoprotien functionalized Pt–NCs with size ∼ 5 nm in a spherical shape. A minimum inhibitory concentration of 12.5 μM of *salmonella typhi* was found using Pt–NCs, and the hemolytic test showed low cytotoxicity at 100 μM [27]. Zhang et al., produced a biosensor to detect staphylococcal enterotoxin A (SEA) using DNA/Ag NCs-PPy. The SEA concentration was in a range of 0.5–1000 ng mL^−1^with the detection limit of 0.3393 ng mL^−1^. This sensor was used in milk samples with the highest recovery efficiency of 94.56% [29]. Yao et al., synthesized silver NCs and detected a food-borne pathogen, i.e., *Staphylococcus aureus*, using Ag NCs. The *S. aureus* ranges from 10 to 10^6^ cfu mL^−1^, and the limit of detection of 4.9 CFU mL^−1^ with the recovery of 85.6%–103.7% [64]. Chen and his coworkers made a dual recognition with aptamer and antibiotic to detect *Staphylococcus aureus* (SA) using Vancomycin-Au-NCs. The sensitivity of Vancomycin-AuNCs detection assay towards SA is in the range of 3x10^8^ cfu. mL^−1^ with the detection limit of 16 CFU mL^−1^. Tan et al. [65], used eggshell membrane-AuNCs (AuNCs-ESM) for the detection of pathogen Staphylococcal enterotoxin B (SE-B) within the concentration range of 0.4–20 ng mL^−1^ with a better limit of detection of 0.12 ng mL^−1^ in the flour, corn and rice-based food samples. The same group synthesized the chitosan-AuNCs to detect three different kinds of bacteria, such as *S. aureus*, *E. coli* and *B. subtilis*. This biosensor had more selectivity towards the *S.aureus* than the *E.coli* and *B. subtilis* with the detection of *S.aureus* as 4 × 10^2^ CFU/mL. Khan et al. [26], detected the T-2 (trichothecenes mycotoxin) using aptamer functionalized Ag NCs (apt-Ag NCs) by fluorescent resonance energy transfer detection method. The T-2 range was found 0.005–500 ng mL^−1^ and 0.93 pg mL^−1^ in maize and wheat samples and LOD with improved recovery efficiency ranged from 89.46% to 102.08% and 90.41% to 107.75% [38]. Ariani and his coworkers detected *Listeria monocytogenes* by MPA-AuNCs with fluorescent technique. In a 10-μL sample, the detection of bacteria was sensed quickly with LOD of 2000 CFU (Table 1).

**Table 1:** The Au based sensors detection of food contaminants.

AUNCs/AUNPs	Stabilizer/Method	Target Food Contaminants	Linear Range	Detection Level	References
AuNCs	Vancomycin	*Staphylococcus aureus*	32-10^8^ cfu/mL	16 cfu/mL	[64]
AuNCs	3- mercaptopropionic acid	*Listeria monocytogenes*	2 × 10^5^ -10^6^	2 × 10^5^ cfu/mL	[38]
AuNCs	Acetylcholinesterase	Organophosphate flame retardants	50–1000 ng L^−1^	30 ng L^−1^	[66]
AuNCs	Eggshell membrane	Staphylococcal enterotoxins B	0.4–20 ng mL^−1^	0.12 ng mL^−1^	[65]
AuNCs–MnO_2_	Composite Based	Carbamate Insecticide	0-50 μL^−1^	0.125μgL^−1^	[67]
AuNCs	chicken egg white	Organophosphorus Pesticides	0.25–50.0 ng mL^−1^	0.1 ng mL^−1^	[45]
AuNCs	BSA- Capped	Olaquindox	1.0 μg kg^−1^ to 150 μg kg^−1^	0.68 μg kg^−1^	[68]
AuNCs @SiO2-MIPs	Composite Based - sol-gel process	Erythromycin	0.1–11.9 μM	12 nM	[69]
AuNCs	His-AUNCs/Lys-AUNCs/RNase-AUNCs	2-methyl-4-chlorophenoxyacetic acid	110–180 nM, 100–170 nM and 90–160 nM	21.87/12.97/9.26 nM	[70]
AuNCs	Histidine and glutathione	Pb^2+^	5.0–1000 nM	1.0 nM	[16]
AuNPs	quinone-methide-type	Fluoride ion	120 μM to 1.5 mM	120 μM	[71]
QD-AuNPs	Mercaptophenyl-boronic acid modified AuNPs (AuNPs-MPBA)	Fluoride ion	5.0–45 mM	50 nM	[72]
AuNCs	Citrate	Cyanide	0.1–300 μM	1.0 × 10^−7^ M	[64]
AuNCs	Lysozyme	Cyanide	5.00 × 10^6^ M − 1.20 × 10^4^ M	1.9 × 10^−7^ M	[51]
AuNPs	Polyethylene glycol	Arsenic	5–20 ppb	5 ppb	[73]
AuNPs	Europium	Arsenic	NA	≤10.0 ppb	[39]
AuNPs	Glutathione	Arsenic	NA	0.11 ppb	[73]
AuNCs	Amylase	Deltamethrin and Glutathione	0.01–5 μM and 0.05–5 μM	6 and 10 nM	[41]
AuNCs	Aprotinin	Trypsin	0–150 μg/ml	10.18 μg mL^−1^	[74]
AuNCs	Bovine serum albumin (BSA)	Cysteine and Homocysteine	0.0057–5 μM and 8–25 μM	9 and 12 nM	[75]
AuNCs	Bovine serum albumin (BSA)	Hg^2+^ and Cyhalothrin	0.00075–5.0 μM and 0.01–10 μM	0.0003 and 0.0075 μM	[48]
AuNCs	Chicken egg white	Antioxidant compounds	0.02–500 μM	0.9 μM	[46]
AuNCs	Ovalbumin	Cu^2+^	5.0–100.0 μmol/L	640.0 nM	[47]
AuNCs	Soybean protein	Bismerthiazol	5–100 μg mL^−1^	5 μg mL^−1^	[50]
AuNCs	Trypsin	Hg^2+^	50–600 nM	50 ± 10 nM	[76]
AuNCs	Amino acid - 6-aza-2-thiothymine and L-arginine	clenbuterol and ractopamine	0.005–4.0 μg L^−1^ and 0.025–4.0 μg L^−1^	0.003 and 0.023 μg L^−1^	[77]
AuNCs	Glutathione	Thiram - Fungicide	0.05–0.5 μg mL^−1^	0.025 μg mL^−1^	[78]
AuNCs	BSA	Hg^2+^	1.0 × 10^−5^ to 5.0 × 10^−13^ M	1.8 × 10^−13^ M	[79]
AuNPs	citrate-mediated reduction – Fren’s Method	Cu^2+^	1.0–17.0 nM	0.37 nM	[80]
AuNCs	Chitosan	Staphylococcal enterotoxin B	NA	1.0 × 10^−12^ g/mL	[81]
AuNCs	Glutathione	Melamine	1.0 × 10^−4^ – 8.0 × 10^−3^ M	2.8 × 10^−5^ M	[43]
AuNCs	BSA/MPA-AUNCs	NO_2_^−^	5–30 μM	0.7 μM	[49]

### 3.2 Pesticides

Pesticides are the chemical substances that protect agricultural products from insects, pests or weeds. These chemical substances when accumulated in water, soil, air and food, leads to more severe health issues in animals and humans. Hence, adequate measures must be taken to control sense and detect the pesticides using MNCs [21]. Hu et al., prepared a poly (sodium-p-styrene sulfonate)-D-penicillamine stabilized AgNCs (PSS-DPA-AgNCs) to detect the food flavors such as 2-Mercapto-3-butanol (2-M-3-B), 3-Mercapto-2-butanone (3-M-2-B) and silicate (SiO_3_^2−^). A simple fluorometric technique was used in 2-M-3-B/3-M-2-B assay in the range of 0.33–90.0/0.33–80.0 μM and a 74/250 nM detection limit. Similarly, for SiO_3_^2−^ assay, corresponding data were 3.33–100.0 μM and 278 nm [82]. A CTAB-CuNCs based chemosensor developed for the detection of dithiocarbamates (non-systemic pesticicdes) by integrating both fluorescent and colorimetric techniques (Table 2) [41]. Bhamore et al., detected deltamethrin and glutathione using amylase-AuNCs by “turn off” fluorescence quenching mechanism. Detection of deltamethrin and GSH were done under fluorescent probe in the range of (0.01–5 μM) and (0.05–5 μM) with good linearity and LOD of 6 and 10 nm [83]. Sun et al., developed an electrochemical immunosensor to detect carbofuran pesticide using Au NPs and Prussian blue-multiwalled carbon nanotubes-chitosan (PB-MWCNTs-CTS) nanocomposite film. The system provided a wide linear range between 0.1 and 1 μg/mL with a low detection limit of 0.021 ng mL^−1^ [45]. Yan and his colleagues developed the assays of organophosphorus pesticides (OPs) using tyrosinase-gold NCs (TYR-AuNCs) by fluorometric technique. Rapid detection of OPs by fluorescence (paraoxon as a model) with a LOD of 0.1 ng mL^−1^ also provides excellent sensitivity [67]. Yan and his coworkers used AuNCs–MnO_2_ for the detection of carbamate pesticide by Fluorescence resonance energy transfer (FRET) effect. A dual-output assay, via color and fluorescence, was used to detect carbaryl with LOD of 0.125 μg L^−1^ [84]. The BSA-CuNCs used for the detection of paraoxon organophosphates by enzyme-free electrochemical biosensor technique. BSA-CuNCs were used as redox-active on the surface electrode with SWCNT and glassy carbon electrodes. The reduction peak current *vs.* paraoxon concentration was linear over the range from 50 nM to 0.5 μM and from 0.5 to 35 μM respectively, with a LOD of 12.8 nM [78]. Zhao et al., used GSH-AuNCs to sense trace amounts of thiram residues in agriculture and food samples via an aggregation-induced emission enhancement (AIEE) mechanism. The detection assay showed an LOD of 0.05 μg mL^−1^ (Table 1).

**Table 2:** Sources, Targets and Detection range of Cu.

CuNCs	Stabilizer/Method	Target Food Contaminants	Linear Range	Detection Level	References
CuNCs	Glutathione	Fe^3+^	1–100 μM	0.3 μM	[36]
CuNCs	Poly(vinylpyrrolidone)	Trinitrophenol	0–30 μM	3.91 × 10^−7^ M	[19]
CuNCs	polyethylene imine	2,4,6-trinitrotoluene	0–9 nM	14 pM	[20]
CuNPs	Ascorbic acid	Urea	0.25–5 mM	0.01 mM	[85]
CuNCs	BSA and single-walled carbon nanotubes	Organophosphates	50 nM to 0.5 μM and 0.5–35 μM	12.8 nM	[84]
CuNCs	Thiosalicylic acid, and cysteamine	Chromium	0.1–1000 mM	0.03 mM	[86]

**Table 3:** Sources, Targets and Detection range of various Nanoclusters.

Metal Nanocluster	Stabilizer/Method	Target Food Contaminants	Linear Range	Detection Level	References
AgNCs	poly methacrylic acid (PMAA)	Microbes	NA	NA	[23]
AgNCs	BSA- IgY Ab	*Listeria monocytogenes*	10 to 10^6^ cfu mL^−1^	10 cfu/mL^−1^	[24]
AgNCs	DNA	Acetamiprid	5–200 μmol/L	2.8 μmol/L	[25]
AgNCs	one pot synthesis of aptamer	T-2 Mycotoxin	0.005–500 ng mL^−1^	0.93 pg mL^−1^	[26]
AgNCs	DNA	Staphylococcal enterotoxin A	0.5–1000 ng mL^−1^	0.3393 ng mL^−1^	[27]
AgNCs	DNA	OTA and AFB_1_	0.001 ng mL^−1^ to 0.05 ng mL^−1^	0.2 pg mL^−1^ and 0.3 pg mL^−1^	[28]
AgNCs	Proteins	*Staphylococcus aureus*	10 to 10^6^ cfu mL^−1^	4.9 cfu/mL	[29]
AgNCs	DNA	Oxytetracycline	0.5 nM–100 nM	0.1 nM	[30]
AgNCs	poly(sodium-p-styrenesulfonate)-enhanced and D-penicillamine stabilized argentum	2-Mercapto-3-butanol/3-Mercapto-2-butanone/silicate	0.33–90.0/0.33–80.0 μM and a 74/250 nM	72/250/278 nM	[21]
Platinum NCS	chicken egg white	Carbidopa	5.0–35 μM	1.71 μM	[31]
Platinum NPs	Proteins	*Salmonella typhi*	NA	NA	[32]
Fe–NCs	Protein	*Salmonella typhimurium*	3.0 × 10^2^ to 3.0 × 10^6^ CFU/mL	14 CFU/mL	[33]
Immune invertase- NCs	co-precipitation	*E. coli*	79 CFU/mL	NA	[34]

### 3.3 Metal ions

**Fig. 6:**
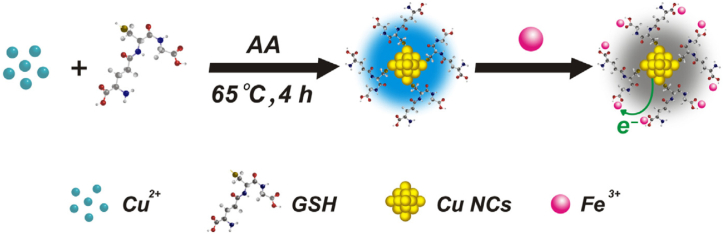
General Mechanism of CuNCs for Fe^3+^ sensing Copyright from Ref. [36].

Lin et al., prepared bi-ligand CuNCs to detect hexavalent chromium in water [86]. A wide linear range of 0.1–1000 mM with a lower LOD of 0.03 mM was obtained in chromium assay. The recoveries of samples were between 98.3 and 105.0% indicating a better repeatability. Huang et al., used CuNCs for the detection of Fe ^3+^ ion in water [36]. In real water sample assay, the fluorescence of CuNCs was linearly quenched upon the increasing Fe^3+^ concentrations in the range of 1–100 μM, with the LOD of 0.3 μM (Fig. 6) (Table 2). A biological sensor was developed based on AuNCs-GO (gold NCs/graphene oxide) and investigated to detect Hg^2+^ in the water sample. In an actual water sample, the fluorescence peak intensity linearly decreases with increasing Hg^2+^ concentration in the range of 1.0 × 10^−5^ to 5.0 × 10^−13^ M with a detection limit of 1.8 × 10^−13^ M [79].

### 3.4 Antibiotic drugs

**Fig. 7:**
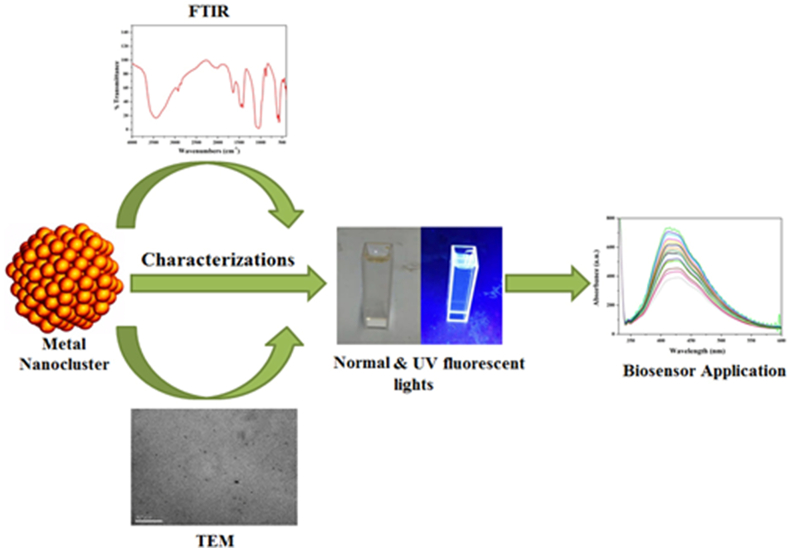
Representation of MNCs, characterization and application.

The substance or compounds that could kill/inhibit the growth of microorganisms were classified into drugs and antibiotics (Fig. 7). These compounds had been widely used in animal husbandry or agriculture as a growth promoter or inhibitor for the microorganisms. However, the usages of these compounds are frequently increased; there should be a proper control towards the usage and detection of these materials towards the environmental benefit [77]. The AuNCs-immunoassay biosensor has been reported for the sensing of clenbuterol and ractopamine. The sensing assay exhibited good linear relationship over the concentrations range of clenbuterol and ractopamine were 0.06 and 0.32 μg L^−1^, respectively, with LOD of 0.003 and 0.023 μg L^−1^ [87]. A CuNCs-BSA was used to identify the kojic acid by fluorescence technique. This biosensor exhibited an excellent detection of the kojic acid in the range of 0.2 μM–50 μM, with LOD of 0.07 μM [50]. Cheng and his colleagues successfully detected bismerthiazol by using soybean protein-capped AuNCs. High selectivity and sensitivity in the concentration range of 5–100 μg mL^−1^ with as low as 5 μg mL^−1^ of bismerthiazol [30]. Hosseini et al., prepared a biosensor using DNA aptamer-Ag NCs to detect oxytetracycline (OTC) by fluorescence technique. The DNA-AgNCs were quenched linearly in the range of 0.5 nM–100 nM with a LOD of 0.1 nM [46]. detected a series of antioxidant compounds using CEW-AuNCs by measuring the Cu (II)-induced prooxidant activity (Table 1). Borse et al. [31], used CEW-Pt NCs for the detection of carbidopa assay. The carbidopa acts as a quencher, resulting in the range of 5.0–35 μM and exhibited a linear response with the LOD of 1.71 μM.

## 4 Advantages and challenges associated with MNCs -based fluorescence sensors in food analysis

Many fluorescent nanomaterials such as the semiconducting QDs and MNCs have considerably less solubility and stability in complex food matrices and biological mediums. Researchers have used several surface modification strategies to afford biocompatibility and stability to address this shortfall. In contrast, MNCs are highly photostable and less toxic. It has been found that MNCs showed high stability and sensitivity toward target analytes in complex food matrices such as milk, fruits and meat samples. As discussed in the previous section, surface passivation of MNCs with suitable capping agents affords unique fluorescence properties. When ligand-capped MNCs are used to detect various analytes, their fluorescence behavior shows an excellent response. The sensing effectiveness of fluorescent nanomaterials is influenced by surface functionalization features such as charge and hydrophobicity, in addition to their inherent photophysical capabilities. Moreover, the interfacial interactions of nanomaterials against target analytes are predominantly controlled by surface functionalization. For example, small organic molecules with aromatic structures undergo strong interaction with the MNCs through electrostatic interaction, π–π stacking, or hydrogen bonding, thereby resulting in FRET or fluorescence quenching. Inspite of possessing significant advantages, MNCs -based fluorescence assays have some disadvantages as well. For instance, factors such as low quantum yield and the unclear relationship between the surface chemistry and physicochemical properties have restricted the performance of MNCs fluorescence sensing assays. The up-conversion and two-photon fluorescence properties of MNCs remain unexplored till now, and it must be utilized for food-based assays. It can be concluded that there is still plenty of space available for future researches in the development of novel MNCs -based fluorescence sensing strategies toward food analysis.

The functionality as well as the accuracy of the results are both key factors to consider while preparing food samples. The pretreatments are carried out based on the food matrix and sensor properties. Liquid foods (drinking water or beverages) are generally easier to pre-treat than solid foods, because of the complexity of food substrate (meat or cereal). On the other hand, plant-based foods (fruits and vegetables) are, easier to prepare than animal-based foods (fish or chicken) [88,89]. According to these research, liquid foods can be measured directly without any treatment [33]. On the other hand, Solid meals require multiple procedures to extract analytes, including crushing, combining with solvents, and centrifugation, before the built-in sensors can be tested. For 220 unique foods, numerous innovative preparation methods have recently been investigated. For example, pre-degassing, was utilized in the preparation of beer [90]. To prevent interfering with test results, the newer procedure includes extracting the target with an organic solvent and then evaporating the solvent [91]. These pretreatment techniques are gradually improving, resulting in more efficient operations and processes. Most of the reported MNCs -based fluorescence real sample analysis follows a simple pretreatment process such as drying, grinding, centrifugation, and filtration. In recent years, various extraction techniques such as solid-phase microextraction, dispersive micro-solid phase extraction, stir bar solid-phase extraction, dispersive solid-phase extraction, magnetic solid-phase extraction etc., have been developed for effective extraction. It is to be noted that each extraction technique is unique in its functioning and extraction ability with inherent advantages and limitations. As a result, the choice of the extraction method has to be decided according to the properties of the target analytes and sample matrices.

## 5 Future and outlooks

The MNCs (M: Au, Ag, Cu etc.,) based biosensors have been reviewed to detect various elements such as microorganisms, pesticides, drugs-antibiotics, metal ions, amino acids and food additives. Many detection techniques have been discussed, such as fluorescent, colorimetric, SERS detection and some newer techniques. In most of the research, the MNCs-based biosensor shows better sensitivity, reliability, specificity and reproducibility. However, many researches still need to be done to solve issues that are crucial for MNCs-based biosensor, such as single step, simple method, minimal washing, fast results, low cost, and homogeneous assay. In MNCs based biosensor with the target analysis, the quality should be maintained qualitatively and quantitatively with binding activity. The MNCs-based biosensor cannot maintain consistent binding activity. Therefore, the standardizing detection techniques will be complex. The cross-linking principle in MNCs will help introduce NCs ' novel properties to improve and promote food safety.

In general, a simple, fast detecting, low cost-based MNCs biosensor needs to be commercialized. The complex synthesis technique and expensive reagents used for the synthesis of MNCs, need to be replaced by a simple and rapid synthesis. Still, many biomolecules in the food contaminants cannot be detected and several steps are required to detect the contaminants. Therefore, research in the field of biosensors needs to be further advanced. The MNCs based biosensor has a certain detection limit, binding activity and it can’t be compromised by storage and cost in real applications. Furthermore, research on MNCs-based biosensors is needed to develop more commercial and viable products for the detection of microorganisms to protect food and the environment.

## Author contribution statement

All authors listed have significantly contributed to the development and the writing of this article.

## Data availability statement

Data included in article/supp. material/referenced in article.

## References

[1] Li H., Ahmad W., Rong Y., Chen Q., Zuo M., Ouyang Q., Guo Z. (2020). Designing an aptamer based magnetic and upconversion nanoparticles conjugated fluorescence sensor for screening Escherichia coli in food. Food Control.

[2] Bayles A., Carretero-Palacios S., Calió L., Lozano G., Calvo M.E., Míguez H. (2020). Localized surface plasmon effects on the photophysics of perovskite thin films embedding metal nanoparticles. J. Mater. Chem. C.

[3] H R C., Schiffman J.D., Balakrishna R.G. (2018). Quantum dots as fluorescent probes: synthesis, surface chemistry, energy transfer mechanisms, and applications. Sensor. Actuator. B Chem..

[4] Zhang L., Wang E. (2014). Metal nanoclusters: new fluorescent probes for sensors and bioimaging. Nano Today.

[5] Kong L., Chu X., Ling X., Ma G., Yao Y., Meng Y., Liu W. (2016). Biocompatible glutathione-capped gold nanoclusters for dual fluorescent sensing and imaging of copper(II) and temperature in human cells and bacterial cells. Microchim. Acta.

[6] Liu M., Zhang X., Yang B., Li Z., Deng F., Y-C Y. (2015).

[7] Reta N., Saint C.P., Michelmore A., Prieto-Simon B., Voelcker N.H. (2018). Nanostructured electrochemical biosensors for label-free detection of water- and food-borne pathogens. ACS Appl. Mater. Interfaces.

[8] Castillo J., Gáspár S., Leth S., Niculescu M., Mortari A., Bontidean I., Soukharev V., Dorneanu S.A., Ryabov A.D., Csöregi E. (2004). Biosensors for life quality - design, development and applications. Sensor. Actuator. B Chem..

[9] Qu X., Li Y., Li L., Wang Y., Liang J., Liang J. (2015). Fluorescent gold nanoclusters: synthesis and recent biological application. J. Nanomater..

[10] Yu W.W., Chang E., Drezek R., Colvin V.L. (2006). Water-soluble quantum dots for biomedical applications. Biochem. Biophys. Res. Commun..

[11] S. Kailasa, S. Borse, J. Koduru, Z.M.-T. in Environmental, undefined 2021, Biomolecules as promising ligands in the synthesis of metal nanoclusters: Sensing, bioimaging and catalytic applications, Elsevier. (n.d.). https://www.sciencedirect.com/science/article/pii/S2214158821000271 (accessed May 18, 2022).

[12] Khan I.M., Niazi S., Yue L., Zhang Y., Pasha I., Iqbal Khan M.K., Akhtar W., Mohsin A., Chughati M.F.J., Wang Z. (2022). Research update of emergent gold nanoclusters: a reinforced approach towards evolution, synthesis mechanism and application. Talanta.

[13] Arumugam S.S., Xuing J., Viswadevarayalu A., Rong Y., Sabarinathan D., Ali S., Agyekum A.A., Li H., Chen Q. (2020). Facile preparation of fluorescent carbon quantum dots from denatured sour milk and its multifunctional applications in the fluorometric determination of gold ions, in vitro bioimaging and fluorescent polymer film. J. Photochem. Photobiol. Chem..

[14] Wang S., Wang X., Zhang Z., Chen L. (2015). Highly sensitive fluorescence detection of copper ion based on its catalytic oxidation to cysteine indicated by fluorescein isothiocyanate functionalized gold nanoparticles. Colloids Surf. A Physicochem. Eng. Asp..

[15] He W., Gui R., Jin H., Wang B., Bu X., Fu Y. (2018). Ratiometric fluorescence and visual imaging detection of dopamine based on carbon dots/copper nanoclusters dual-emitting nanohybrids. Talanta.

[16] Bhamore J.R., Gul A.R., Chae W.S., Kim K.W., Lee J.S., Park H., Kailasa S.K., Park T.J. (2020). One-pot fabrication of amino acid and peptide stabilized gold nanoclusters for the measurement of the lead in plasma samples using chemically modified cellulose paper. Sensor. Actuator. B Chem..

[17] Farzin L., Shamsipur M., Samandari L., Sadjadi S., Sheibani S. (2020). Biosensing strategies based on organic-scaffolded metal nanoclusters for ultrasensitive detection of tumor markers. Talanta.

[18] Mei L., Teng Z., Zhu G., Liu Y., Zhang F., Zhang J., Li Y., Guan Y., Luo Y., Chen X., Wang Q. (2017). Silver nanocluster-embedded zein films as antimicrobial coating materials for food packaging. ACS Appl. Mater. Interfaces.

[19] Li Y., Feng L., Yan W., Hussain I., Su L., Tan B. (2019). PVP-templated highly luminescent copper nanoclusters for sensing trinitrophenol and living cell imaging. Nanoscale.

[20] Aparna R.S., Anjali Devi J.S., Sachidanandan P., George S. (2018). Polyethylene imine capped copper nanoclusters- fluorescent and colorimetric onsite sensor for the trace level detection of TNT. Sensor. Actuator. B Chem..

[21] Hu Y., Li Y., Liao Y., Jiang X., Cheng Z. (2020). Poly(sodium-p-styrenesulfonate)-enhanced fluorescent silver nanoclusters for the assay of two food flavors and silicic acid. Food Chem..

[22] Lewis J.S., Barani Z., Magana A.S., Kargar F. (2019).

[23] Mei L., Teng Z., Zhu G., Liu Y., Zhang F., Zhang J., Li Y., Guan Y., Luo Y., Chen X., Wang Q. (2017). Silver nanocluster-embedded zein films as antimicrobial coating materials for food packaging. ACS Appl. Mater. Interfaces.

[24] Liu Y., Wang J., Song X., Xu K., Chen H., Zhao C., Li J. (2018). Colorimetric immunoassay for Listeria monocytogenes by using core gold nanoparticles, silver nanoclusters as oxidase mimetics, and aptamer-conjugated magnetic nanoparticles. Microchim. Acta.

[25] B.M.L.X.Y. YaLi, Sensitive detection of acetamiprid silver nanoclusters fluorescent probes based on DNA protection., (n.d.). https://www.cabdirect.org/cabdirect/abstract/20193263431 (accessed December 26, 2020).

[26] Khan I.M., Zhao S., Niazi S., Mohsin A., Shoaib M., Duan N., Wu S., Wang Z. (2018). Silver nanoclusters based FRET aptasensor for sensitive and selective fluorescent detection of T-2 toxin. Sensor. Actuator. B Chem..

[27] Zhang X., Khan I.M., Ji H., Wang Z., Tian H., Cao W., Mi W. (2020). A label-free fluorescent aptasensor for detection of staphylococcal enterotoxin A based on aptamer-functionalized silver nanoclusters. Polymers.

[28] Zhang J., Xia Y.K., Chen M., Wu D.Z., Cai S.X., Liu M.M., He W.H., Chen J.H. (2016). A fluorescent aptasensor based on DNA-scaffolded silver nanoclusters coupling with Zn(II)-ion signal-enhancement for simultaneous detection of OTA and AFB1. Sensor. Actuator. B Chem..

[29] Yao S., Zhao C., Liu Y., Nie H., Xi G., Cao X., Li Z., Pang B., Li J., Wang J. (2020). Colorimetric immunoassay for the detection of Staphylococcus aureus by using magnetic carbon dots and sliver nanoclusters as o-phenylenediamine-oxidase mimetics. Food Anal. Methods.

[30] Hosseini M., Mehrabi F., Ganjali M.R., Norouzi P. (2016).

[31] Borse S., Murthy Z.V.P., Kailasa S.K. (2020). Chicken egg white mediated synthesis of platinum nanoclusters for the selective detection of carbidopa. Opt. Mater..

[32] Subramaniyan S.B., Ramani A., Ganapathy V., Anbazhagan V. (2018). Preparation of self-assembled platinum nanoclusters to combat Salmonella typhi infection and inhibit biofilm formation. Colloids Surf. B Biointerfaces.

[33] Zhang H., Xue L., Huang F., Wang S., Wang L., Liu N., Lin J. (2019). A capillary biosensor for rapid detection of Salmonella using Fe-nanocluster amplification and smart phone imaging. Biosens. Bioelectron..

[34] Huang F., Zhang H., Wang L., Lai W., Lin J. (2018). A sensitive biosensor using double-layer capillary based immunomagnetic separation and invertase-nanocluster based signal amplification for rapid detection of foodborne pathogen. Biosens. Bioelectron..

[35] Liu X., Astruc D. (2018). Atomically precise copper nanoclusters and their applications. Coord. Chem. Rev..

[36] Huang H., Li H., Feng J.J., Feng H., Wang A.J., Qian Z. (2017). One-pot green synthesis of highly fluorescent glutathione-stabilized copper nanoclusters for Fe3+ sensing. Sensor. Actuator. B Chem..

[37] Gayen C., Basu S., Goswami U., Paul A. (2019). Visible light excitation-induced luminescence from gold nanoclusters following surface ligand complexation with Zn2+ for daylight sensing and cellular imaging. Langmuir.

[38] Hossein-Nejad-Ariani H., Kim T., Kaur K. (2018).

[39] Nath P., Priyadarshni N., Chanda N. (2018). Europium-coordinated gold nanoparticles on paper for the colorimetric detection of arsenic(III, V) in aqueous solution. ACS Appl. Nano Mater..

[40] Zheng J., Zhang C., Dickson R.M. (2004). Highly fluorescent, water-soluble, size-tunable gold quantum dots. Phys. Rev. Lett..

[41] Bhamore J.R., Jha S., Singhal R.K., Murthy Z.V.P., Kailasa S.K. (2019). Amylase protected gold nanoclusters as chemo- and bio- sensor for nanomolar detection of deltamethrin and glutathione. Sensor. Actuator. B Chem..

[42] Dai R., Deng W., Hu P., You C., Yang L., Jiang X., Xiong X., Huang K. (2018). One-pot synthesis of bovine serum albumin protected gold/silver bimetallic nanoclusters for ratiometric and visual detection of mercury. Microchem. J..

[43] Kalaiyarasan G., Anusuya K., Joseph J. (2017). Melamine dependent fluorescence of glutathione protected gold nanoclusters and ratiometric quantification of melamine in commercial cow milk and infant formula. Appl. Surf. Sci..

[44] Guo Y., Amunyela H.T.N.N., Cheng Y., Xie Y., Yu H., Yao W., Li H.W., Qian H. (2020). Natural protein-templated fluorescent gold nanoclusters: syntheses and applications. Food Chem..

[45] Yan X., Li H., Hu T., Su X. (2017). A novel fluorimetric sensing platform for highly sensitive detection of organophosphorus pesticides by using egg white-encapsulated gold nanoclusters. Biosens. Bioelectron..

[46] Akyüz E., Şen F.B., Bener M., Başkan K.S., Tütem E., Apak R. (2019). Protein-Protected gold nanocluster-based biosensor for determining the prooxidant activity of natural antioxidant compounds. ACS Omega.

[47] Chen Y., Qiao J., Liu Q., Qi L. (2018). Ovalbumin-stabilized gold nanoclusters with ascorbic acid as reducing agent for detection of serum copper. Chin. Chem. Lett..

[48] Bhamore J.R., Jha S., Basu H., Singhal R.K., Murthy Z.V.P., Kailasa S.K. (2018). Tuning of gold nanoclusters sensing applications with bovine serum albumin and bromelain for detection of Hg2+ ion and lambda-cyhalothrin via fluorescence turn-off and on mechanisms. Anal. Bioanal. Chem..

[49] Deng H.H., Huang K.Y., Zhang M.J., Zou Z.Y., Xu Y.Y., Peng H.P., Chen W., Hong G.L. (2020). Sensitive and selective nitrite assay based on fluorescent gold nanoclusters and Fe2+/Fe3+ redox reaction. Food Chem..

[50] Cheng Y., Zhang Y., Pei R., Xie Y., Yao W., Guo Y., Qian H. (2018). Fast detection of bismerthiazol in cabbage based on fluorescence quenching of protein-capping gold nanoclusters. Anal. Sci..

[51] Lu D., Liu L., Li F., Shuang S., Li Y., Choi M.M.F., Dong C. (2014). Lysozyme-stabilized gold nanoclusters as a novel fluorescence probe for cyanide recognition. Spectrochimica Acta - Part A: Molecular Biomol. Spectroscopy.

[52] Sun J., Yue Y., Wang P., He H., Jin Y. (2013). Facile and rapid synthesis of water-soluble fluorescent gold nanoclusters for sensitive and selective detection of Ag+. J. Mater. Chem. C.

[53] Mecker L.C., Tyner K.M., Kauffman J.F., Arzhantsev S., Mans D.J., Gryniewicz-Ruzicka C.M. (2012). Selective melamine detection in multiple sample matrices with a portable Raman instrument using surface enhanced Raman spectroscopy-active gold nanoparticles. Anal. Chim. Acta.

[54] Chen L.Y., Wang C.W., Yuan Z., Chang H.T. (2015). Fluorescent gold nanoclusters: recent advances in sensing and imaging. Anal. Chem..

[55] Ebrahiminezhad A., Berenjian A., Ghasemi Y. (2016). Template free synthesis of natural carbohydrates functionalised fluorescent silver nanoclusters. IET Nanobiotechnol..

[56] Huang J., Li Q., Sun D., Lu Y., Su Y., Yang X., Wang H., Wang Y., Shao W., He N., Hong J., Chen C. (2007). Biosynthesis of silver and gold nanoparticles by novel sundried Cinnamomum camphora leaf. Nanotechnology.

[57] Sathishkumar M., Sneha K., Won S.W., Cho C.W., Kim S., Yun Y.S. (2009). Cinnamon zeylanicum bark extract and powder mediated green synthesis of nano-crystalline silver particles and its bactericidal activity. Colloids Surf. B Biointerfaces.

[58] Wilcoxon J.P., Samara G.A., Provencio P.N. (1999). Optical and electronic properties of Si nanoclusters synthesized in inverse micelles. Phys. Rev. B.

[59] Heo S.G., Yang W.S., Kim S., Park Y.M., Park K.T., Oh S.J., Seo S.J. (2021). Synthesis, characterization and non-enzymatic lactate sensing performance investigation of mesoporous copper oxide (CuO) using inverse micelle method. Appl. Surf. Sci..

[60] Wen Z., Lu J., Zhang Y., Cheng G., Huang S., Chen J., Xu R., an Ming Y., Wang Y., Chen R. (2020). Facile inverse micelle fabrication of magnetic ordered mesoporous iron cerium bimetal oxides with excellent performance for arsenic removal from water. J. Hazard Mater..

[61] Arenas-Vivo A., Rojas S., Ocaña I., Torres A., Liras M., Salles F., Arenas-Esteban D., Bals S., Ávila D., Horcajada P. (2021). Ultrafast reproducible synthesis of a Ag-nanocluster@MOF composite and its superior visible-photocatalytic activity in batch and in continuous flow. J. Mater. Chem..

[62] Li Y., Zhou X., Xing Y. (2020). In situ thermal-assisted loading of monodispersed Pt nanoclusters on CdS nanoflowers for efficient photocatalytic hydrogen evolution. Appl. Surf. Sci..

[63] Huang F., Zhang H., Wang L., Lai W., Lin J. (2018). A sensitive biosensor using double-layer capillary based immunomagnetic separation and invertase-nanocluster based signal amplification for rapid detection of foodborne pathogen. Biosens. Bioelectron..

[64] Cheng D., Yu M., Fu F., Han W., Li G., Xie J., Song Y., Swihart M.T., Song E. (2016). Dual recognition strategy for specific and sensitive detection of bacteria using aptamer-coated magnetic beads and antibiotic-capped gold nanoclusters. Anal. Chem..

[65] Tan F., Xie X., Xu A., Deng K., Zeng Y., Yang X., Huang H. (2019). Fabricating and regulating peroxidase-like activity of eggshell membrane-templated gold nanoclusters for colorimetric detection of staphylococcal enterotoxin B. Talanta.

[66] Liu H., Zhu N., Li M., Huang X., Wu P., Hu Z., Shuai J. (2020). Induced fluorescent enhancement of protein-directed synthesized gold nanoclusters for selective and sensitive detection of flame retardants. Sci. Total Environ..

[67] Yan X., Kong D., Jin R., Zhao X., Li H., Liu F., Lin Y., Lu G. (2019). Fluorometric and colorimetric analysis of carbamate pesticide via enzyme-triggered decomposition of Gold nanoclusters-anchored MnO2 nanocomposite. Sensor. Actuator. B Chem..

[68] Peng T., Wang J., Xie S., Yao K., Zheng P., Ke Y., Jiang H. (2019). Label-free gold nanoclusters as quenchable fluorescent probes for sensing olaquindox assisted by glucose oxidase-triggered Fenton reaction. Food Addit. Contam. Part A Chemistry, Analysis, Control, Exposure Risk Assessment.

[69] Zhang Y., Zhou Z., Zheng J., Li H., Cui J., Liu S., Yan Y., Li C. (2017). SiO2-MIP core-shell nanoparticles containing gold nanoclusters for sensitive fluorescence detection of the antibiotic erythromycin. Microchim. Acta.

[70] Nazir K., Ahmed A., Hussain S.Z., Younis M.R., Zaheer Y., Ahmed M., Hussain I., Ihsan A. (2020). Development of gold nanoclusters based direct fluorescence restoration approach for sensitive and selective detection of pesticide. Appl. Nanosci..

[71] Gu J.A., Lin Y.J., Chia Y.M., Lin H.Y., Huang S.T. (2013). Colorimetric and bare-eye determination of fluoride using gold nanoparticle agglomeration probes. Microchim. Acta.

[72] Xue M., Wang X., Duan L., Gao W., Ji L., Tang B. (2012). A new nanoprobe based on FRET between functional quantum dots and gold nanoparticles for fluoride anion and its applications for biological imaging. Biosens. Bioelectron..

[73] Lewis J.S., Barani Z., Magana A.S., Kargar F. (2019).

[74] Gao P., Wu S., Chang X., Liu F., Zhang T., Wang B., Zhang K.Q. (2018). Aprotinin encapsulated gold nanoclusters: a fluorescent bioprobe with dynamic nuclear targeting and selective detection of trypsin and heavy metal. Bioconjugate Chem..

[75] Nebu J., Anjali Devi J.S., Aparna R.S., Aswathy B., Lekha G.M., Sony G. (2019). Potassium triiodide-quenched gold nanocluster as a fluorescent turn-on probe for sensing cysteine/homocysteine in human serum. Anal. Bioanal. Chem..

[76] Kawasaki H., Yoshimura K., Hamaguchi K., Arakawa A.R. (2011). Trypsin-stabilized fluorescent gold nanocluster for sensitive and selective Hg 2+ detection. Anal. Sci..

[77] Peng T., Wang J., Zhao S., Zeng Y., Zheng P., Liang D., Mari G.M., Jiang H. (2018). Highly luminescent green-emitting Au nanocluster-based multiplex lateral flow immunoassay for ultrasensitive detection of clenbuterol and ractopamine. Anal. Chim. Acta.

[78] Zhao X., Kong D., Jin R., Li H., Yan X., Liu F., Sun P., Gao Y., Lu G. (2019). On-site monitoring of thiram via aggregation-induced emission enhancement of gold nanoclusters based on electronic-eye platform. Sensor. Actuator. B Chem..

[79] Xiaofei W., Ruiyi L., Zaijun L., Junkang L., Guangli W., Zhiguo G. (2014). Synthesis of double gold nanoclusters/graphene oxide and its application as a new fluorescence probe for Hg2+ detection with greatly enhanced sensitivity and rapidity. RSC Adv..

[80] Wang S., Wang Y., Zhou L., Li J., Wang S., Liu H. (2014). Fabrication of an effective electrochemical platform based on graphene and AuNPs for high sensitive detection of trace Cu2+. Electrochim. Acta.

[81] Xie X., Tan F., Xu A., Deng K., Zeng Y., Huang H. (2019). UV-induced peroxidase-like activity of gold nanoclusters for differentiating pathogenic bacteria and detection of enterotoxin with colorimetric readout. Sensor. Actuator. B Chem..

[82] Chen S., Wang Y., Feng L. (2020). Specific detection and discrimination of dithiocarbamates using CTAB-encapsulated fluorescent copper nanoclusters. Talanta.

[83] Sun X., Du S., Wang X. (2012). Amperometric immunosensor for carbofuran detection based on gold nanoparticles and PB-MWCNTs-CTS composite film. Eur. Food Res. Technol..

[84] Bagheri H., Afkhami A., Khoshsafar H., Hajian A., Shahriyari A. (2017). Protein capped Cu nanoclusters-SWCNT nanocomposite as a novel candidate of high performance platform for organophosphates enzymeless biosensor. Biosens. Bioelectron..

[85] Deng H.H., Li K.L., Zhuang Q.Q., Peng H.P., Zhuang Q.Q., Liu A.L., Xia X.H., Chen W. (2018). An ammonia-based etchant for attaining copper nanoclusters with green fluorescence emission. Nanoscale.

[86] Lin Y.S., Chiu T.C., Hu C.C. (2019). Fluorescence-tunable copper nanoclusters and their application in hexavalent chromium sensing. RSC Adv..

[87] Gao Z., Su R., Qi W., Wang L., He Z. (2014). Copper nanocluster-based fluorescent sensors for sensitive and selective detection of kojic acid in food stuff. Sensor. Actuator. B Chem..

[88] Wu S., Zhang H., Shi Z., Duan N., Fang C.C., Dai S., Wang Z. (2015). Aptamer-based fluorescence biosensor for chloramphenicol determination using upconversion nanoparticles. Food Control.

[89] Zhang D., Liu H., Geng W., Wang Y. (2019). A dual-function molecularly imprinted optopolymer based on quantum dots-grafted covalent-organic frameworks for the sensitive detection of tyramine in fermented meat products. Food Chem..

[90] Cao Y., Hu X., Zhao T., Mao Y., Fang G., Wang S. (2021). A core-shell molecularly imprinted optical sensor based on the upconversion nanoparticles decorated with Zinc-based metal-organic framework for selective and rapid detection of octopamine. Sensor. Actuator. B Chem..

[91] Yu Z., Ma W., Wu T., Wen J., Zhang Y., Wang L., He Y., Chu H., Hu M. (2020). Coumarin-modified graphene quantum dots as a sensing platform for multicomponent detection and its applications in fruits and living cells. ACS Omega.

